# Association between clozapine-induced hypertriglyceridemia and defenestration of liver sinusoidal endothelial cells

**DOI:** 10.1038/s41398-026-04328-4

**Published:** 2026-08-06

**Authors:** Tetyana Voloshyna, Øyvind V. Svendsen, Karolina Szafranska, Jakub Pospíšil, Eike C. Struck, Elisabet Størset, Robert L. Smith, Espen Molden, David G. Le Couteur, Victoria C. Cogger, Peter A. G. McCourt, Erik S. Dietrichs

**Affiliations:** 1 Vascular Biology Research Group, Department of Medical Biology, UiT The Arctic University of Norway, Tromsø, Norway; 2 Institute for Experimental Medical Research, Oslo University Hospital and University of Oslo, Oslo, Norway; 3 Center for Psychopharmacology, Diakonhjemmet Hospital, Oslo, Norway; 4 Translational Vascular Research Group, Department of Clinical Medicine, UiT The Arctic University of Norway, Tromsø, Norway; 5 The Faculty of Mathematics and Natural Sciences, University of Oslo, Oslo, Norway; 6 ANZAC Research Institute, Concord Hospital, Sydney Local Health District and Faculty of Medicine and Health, University of Sydney, Sydney, NSW Australia; 7 Charles Perkins Centre, University of Sydney, Sydney, NSW Australia

**Keywords:** Schizophrenia, Physiology, Clinical pharmacology

## Abstract

Clozapine is the most effective antipsychotic drug, while also causing the most severe metabolic side effects. The underlying mechanisms of these side effects appear multifactorial and are not entirely understood. We hypothesized that liver sinusoidal endothelial cells (LSEC) dysfunction is linked to the metabolic side effects of clozapine, based on known association between reduced LSEC porosity, hypertriglyceridemia and insulin resistance. Accordingly, we conducted a translational study by assessing the porosity of murine LSECs following clozapine exposure in vitro and correlating serum clozapine concentrations with metabolic parameters in routine human blood samples. The patient data showed a correlation for clozapine serum concentrations with both triglycerides (Spearman’s ρ = 0.47, *P* = 0.00024) and glucose levels (ρ = 0.24, *P* = 0.034), but no correlation with LDL cholesterol (ρ = −0.04, *P* = 0.82) or HDL cholesterol (ρ = −0.22, *P* = 0.088). The in vitro experiments showed a concentration-dependent reduction in murine LSEC porosity (P for trend = 0.00036). Together, these findings support a link between clozapine exposure and reduced LSEC porosity. This represents a promising point of intervention that potentially could expand safe access to clozapine as a highly effective antipsychotic treatment for a broader population, also in individuals at high risk for metabolic and cardiovascular side effects.

## Introduction

Schizophrenia affects over 20 million people worldwide and is among the most disabling psychiatric disorders [1]. In addition to affecting cognition, behaviour, and social functioning, the disease is associated with increased risk of metabolic syndrome [2], a cluster of conditions characterized by abdominal obesity, insulin resistance, dyslipidaemia, and hypertension [3]. While lifestyle factors contribute to this metabolic risk, it is significantly exacerbated by antipsychotic medications, which are known to cause metabolic dysregulation [4]. Consequently, the prevalence of metabolic syndrome is higher in antipsychotic-treated individuals with schizophrenia than in the general population [5, 6]. Although successful antipsychotic treatment may improve lifestyle factors related to metabolic health, the metabolic side effects of these drugs often outweigh such benefits. This, in turn, increases risk for lethal cardiovascular disease, contributing to the 14.5-year reduction in life expectancy observed in this group [7].

For approximately one-third of people with schizophrenia, treatment is further complicated by inadequate response to standard therapy [8]. While clozapine is the most effective antipsychotic and the recommended option for individuals with treatment-resistant illness [9, 10], it is also associated with the highest risk of metabolic syndrome [4, 11]. This limits access to effective treatment for people with an already unacceptable cardiovascular risk profile [12]. The mechanisms driving the metabolic side effects are not fully understood and can only partially be explained by weight gain [13, 14]. This is shown by the fact that GLP-1 analogues successfully induce weight loss in individuals using clozapine but fail to normalize triglyceride and glucose serum levels, indicating a direct and negative effect of clozapine on triglyceride clearance and sensitivity to insulin [15]. Following a comprehensive review of the literature, Pillinger et al. concluded that there is a need for further research on the mechanisms underlying the metabolic side effects of antipsychotic drugs [4]. A deeper understanding of these mechanisms is essential for developing personalized treatment strategies and mitigating potentially lethal complications, including atherogenic effects and myocardial infarction.

Fundamental in this setting, the liver is the primary regulator of metabolism. While several hepatic side effects of clozapine are described, like elevated liver enzymes, hepatitis, and cirrhosis, less is known about the role of the liver in the development of metabolic side effects. One of the important metabolic functions performed by the liver is clearance and synthesis of lipoproteins, containing triglycerides. Specialized liver sinusoidal endothelial cells (LSEC) line the hepatic sinusoids and play a vital role in maintaining metabolic homeostasis [16]. Patent nanopores (50–300 nm in diameter), termed fenestrations, cover the cell surface area and provide effective ultrafiltration of small molecules, including lipoproteins and insulin, between the plasma and the hepatocytes [17]. Ageing [18], liver disease [19], drugs, and toxic substances can cause the loss of fenestrations [17], which has been shown to reduce the transport of triglyceride-rich chylomicrons and insulin, resulting in atherosclerosis and insulin resistance [20, 21]. Impaired LSEC function is thus increasingly recognized as a significant factor in the pathophysiology of metabolic syndrome [18].

We hypothesize that clozapine exposure reduces LSEC porosity, which in turn might impair liver clearance of large lipoproteins and secondarily increase the risk of cardiovascular disease. Elucidating the mechanisms underlying clozapine-induced dyslipidaemia could facilitate the development of targeted interventions to mitigate the metabolic burden of antipsychotic treatment, thereby improving both health and life expectancy of people with schizophrenia.

## Methods

### Patient data

The study was approved by the Hospital Investigational Review Board and the Regional Committee for Medical Research Ethics (#625204). Ethical approval was obtained without the requirement of patient consent because the study was based on already collected samples and existing data retrospectively retrieved from a routine laboratory service.

#### Blood samples

Data from blood samples analysed at Diakonhjemmet Hospital in Norway between 1 January 2005 and 31 December 2023 were used retrospectively in this study. This represents the period during which clozapine and norclozapine serum concentration measurements were offered as routine analyses at the hospital, up to the beginning of data collection for the study. We included all samples from patients aged 18 years or older where therapeutic drug monitoring (TDM) was performed to measure serum concentrations of clozapine and norclozapine in the same sample as one or more of the following metabolic parameters (MPs): triglycerides, HDL, LDL, glucose, and HbA1c. Information on whether the patients were fasting at the time of blood sampling was not available. Only the last available combination of TDM results and each MP for each patient was included.

All patient data used in this study were retrospectively collected and based on routine analyses. Two different analytical methods based on liquid chromatography tandem-mass spectrometry were used during the data inclusion period, the newest was based on ultra-high-performance liquid chromatography-high resolution mass spectrometry (UHPLC-HRMS). Triglycerides, HDL, LDL, and glucose were analysed by photometry, while HbA1c was analysed by immunoturbidimetry.

To ensure that all included samples had TDM concentrations close to trough values, we excluded samples drawn less than 10 h or more than 30 h after the last clozapine dose, and samples for which information about the time since the previous dose was unavailable. We also excluded samples where TDM of more than one antipsychotic drug was requested on the same requisition to avoid any influence from other antipsychotic drugs on the MPs. For TDM results below the lower limit of quantification or above the upper limit of quantification, an estimated value based on extrapolation from the validated concentration interval was used.

#### Maximum hepatic inlet concentration calculations

Measured concentrations represent the total concentrations, including both unbound active drug and drug bound to plasma proteins, which are expected to be inactive. Accordingly, measured serum concentrations do not represent the maximal, active clozapine concentrations that LSEC in treated persons were exposed to. After intestinal absorption of clozapine, the drug enters the portal vein and reaches the liver sinusoids, lined with LSEC. To estimate the drug concentrations to which the patients’ LSECs were exposed, the highest single clozapine dose administered per 24 h for each patient was recorded based on information provided on the requisition. The estimated unbound maximum hepatic inlet concentration (${I}_{{in},\max ,u}$) was calculated based on the formula ${I}_{{in},\max ,u}=f{u}_{p}\left({I}_{{sys}}+\frac{{Dose}\times {F}_{a}\times {F}_{g}\times {k}_{a}}{{R}_{b}\times {Q}_{H}}\right)$ [22], where $f{u}_{p}$ is the unbound fraction of drug in plasma (0.06 for clozapine and 0.1 for norclozapine [23]), ${I}_{{sys}}$ is the systemic concentration of the drug, Dose is the orally administered drug dose, ${F}_{a}\times {F}_{g}$ is the fraction of drug absorbed from the gut into hepatic portal blood (0.95 [24],), ${k}_{a}$ is the absorption rate constant (1.3/h [25],), ${Q}_{H}$ is the mean hepatic blood flow (97 L/h [26],), and ${R}_{b}$ is the blood to plasma ratio (0.9 [27],). Further information about the assumptions for the selected parameters can be found in Supplementary Table 1.

The measured serum concentrations, representing trough systemic concentrations, were used in the calculations instead of peak systemic serum concentrations for three reasons. Firstly, peak concentrations were not measured in the included patients. Secondly, the contribution from systemic circulation to the maximum hepatic inlet concentration is small compared to the contribution from the direct uptake from the gut. Thirdly, the formula used has been developed for regulatory purposes and includes a theoretical worst-case scenario where peak systemic circulation coincides with the time of maximum uptake from the gut. In reality, the contribution from the systemic concentration will be lower than peak systemic concentrations at the time of maximum uptake from the gut. The total error introduced by using trough serum concentrations instead of peak serum concentrations is therefore likely to be smaller than the individual variations in the pharmacokinetic parameters used.

#### Statistical analysis

The statistical analysis of the patient data was conducted in R (version 4.3.2) using RStudio (version 2023.09.1). Correlation was assessed using Spearman’s rank correlation coefficient. A non-parametric method was chosen because the TDM measurements, and several of the MPs, were not normally distributed. Multiple linear regression analyses were performed to adjust for age, sex, and treatment duration, as these variables represent possible confounders influencing both TDM results and MPs. The residuals from the regression analyses were normally distributed. A significance threshold of *P* < 0.05 was used for all analyses. No transformations were applied to the variables prior to analysis.

Based on the previously established knowledge that LSEC defenestration is associated with hypertriglyceridemia and glucose intolerance, we were expecting a specific pattern of associations between the MPs and TDM results (i.e. clozapine/norclozapine being associated with triglycerides, glucose, and HbA1c, but not with HDL or LDL) if our hypothesis of clozapine causing LSEC defenestrations was true [17]. Because the goal of the study was to establish whether this pattern was present in persons using clozapine, rather than to discover any individual associations, multiple comparisons adjustment was not considered relevant.

### In vitro experiments

The full list of reagents and solutions and corresponding suppliers is provided in the supplementary information.

#### Animals

Young male mice (8 - 14 weeks old), (strain C57 Black6JRj) were purchased from Janvier (Germany/France) and housed at the Department for Comparative Medicine at UiT, at standard conditions, given water and chow diet ad libitum. The experimental protocols were approved by the ethics committee of the Local Animal Research Authority at the UiT The Arctic University of Norway (09/22). All experiments were performed in accordance with relevant national and local guidelines and regulations and reported in accordance with ARRIVE guidelines.

Aged male mice (20 to 24 months old), (strain C57 Black6J) were obtained from the Animal Research Centre in Perth, Western Australia. The mice were housed at the ANZAC Research Institute, Sydney, Australia, on a 12-hour light/dark cycle, given water and chow diet ad libitum, and enrichment. The experimental protocols were approved by the ethics committee of the Sydney Local Health District Animal Welfare Committee (AWC # 2022/025 approval 02/2023–01/2026).

In this study, a total of 19 young and 11 aged mice were used. No formal power analysis was performed prior to conducting the experiments. Sample sizes were determined based on ethical considerations and adherence to 3Rs principle (Replacement, Reduction, Refinement), with the goal of minimizing animal use while maintaining scientific relevance and experimental reproducibility. All experiments were repeated in 3-4 biological replicates, and the consistent effects observed across animals and experiments support the robustness of the findings.

#### LSEC isolation and culture

The mice were sacrificed with cervical dislocation prior to liver perfusion and LSEC extraction. Aged mice were anaesthetized with a mixture of 10 mg/kg xylazine and 100 mg/kg ketamine prior to sacrifice. LSEC were extracted as described by Elvevold et al [28]. Briefly, livers were perfused and digested with Liberase™ TM Research grade (Sigma-Aldrich, St. Louis, US). The hepatocytes were separated and removed by differential centrifugation, while LSEC were further isolated using immune magnetic cell sorting, selecting for CD-146 positive cells (Miltenyi Biotec, Bergisch Gladbach, Germany).

Purified LSEC were seeded on human fibronectin coated wells (0.2 mg/mL) and cultured in serum free RPMI 1640 supplemented with 10 000 U/mL penicillin, 10 mg/mL streptomycin (Cat. no. P4333, Sigma-Aldrich) at 37 °C in 5% CO_2_ and 20% O_2_. To allow attachment to the substrate, LSEC were incubated for 1 h prior to media change that removes non-viable cells. Then, the cells were incubated for one to two hours prior to treatment with clozapine and norclozapine (Sigma-Aldrich Chemie, Schnelldorf, Germany). Seeding and treatment information is described for each assay.

#### LSEC ultrastructure determined with scanning electron microscopy

Mouse LSEC were seeded at a density of 1.8 × 10^5^ cells/cm^2^ on fibronectin coated chambered cover glass (CS16-CultureWell™ Removable Chambered Coverglass, Grace Bio-labs, OR, USA) and treated with matching concentrations (0, 100, 1000, 10 000, 25 000, 50 000, 75 000, 100 000 nmol/L) of clozapine or norclozapine for 1 h prior to fixation with 4% formaldehyde and 1% glutaraldehyde in PHEM buffer (PIPES, HEPES, EGTA, MgSO_4_ – pH 7). All experiments included an untreated control group. Identical experiments were performed on LSEC isolated from young and aged mice. No randomization of treatment placement order or control group placement order was performed, and the experimenters were not blind to group assignments. For the porosity studies, all experiments were repeated in three young and three to four old mice for clozapine and norclozapine.

The sample preparation method for scanning electron microscopy (SEM) included one hour post-fixation with freshly made and filtered (0.2 µm) 1% tannic acid in PHEM buffer, then one hour in 1% OsO_4_ in deionized water and dehydration with graded series of ethanol (30%, 60%, 90%, for five minutes and 100% four times for five minutes) before chemical drying with hexamethyldisilazane (HMDS) (Sigma-Aldrich, Mannheim, Germany), (twice for two minutes).

The specimens were sputter coated with 10 nm Au/Pd and scanned using the Zeiss Gemini 300 scanning electron microscope. The samples were not randomised prior to image acquisition, and users were not blind towards the treatment groups assessed while imaging. However, cells were chosen at random from three or more different regions of the well, with at least five cells from each region, yielding 15–20 images per treatment group per animal. In total, 50–90 images were taken per treatment group. Damaged cells were excluded from the selection.

For automated segmentation of fenestrations in SEM images of LSECs, we trained a custom model using the StarDist framework, which represents objects as star-convex polygons to capture their precise shape [29, 30]. The model, based on a U-Net convolutional architecture, was trained using dataset of 22 manually annotated training images and 11 validation images. This approach enabled robust identification of individual fenestrations, providing segmentation masks that were subsequently used to quantify LSEC porosity (total area of fenestrations per cell area – %), and fenestration frequency (number of fenestrations per cell area – No./µm^2^). The segmentation of fenestration was automated and thus unbiased towards the treatment groups. Prior to quantification of fenestration porosity, the segmentation masks of each cell of all treatment groups were qualitatively validated to ensure that only fenestrations were segmented.

#### Viability study

LSEC were seeded on fibronectin coated 96-well plates at a cell density of 1.5 × 10^5^ cells/cm^2^ and treated with matched concentrations (0-100-1000-10 000-25 000-50 000-75 000-100 000 nmol/L) of clozapine or norclozapine. All experiments included an untreated control group for each animal, and cell-free wells for background control. No randomization of treatment placement order or control group placement order was performed, and the viability study was performed unblinded. All wells were incubated with 10% resazurin solution in culture media for three hours following a 30-minute-long pre-treatment with clozapine or norclozapine. The readouts were performed after three hours of incubation. Viability studies were performed on freshly isolated LSEC from nine young and four old mice to assess the effect of clozapine, and on four young and three old mice to assess the effect of norclozapine.

#### Endocytosis assay

For quantitative studies of endocytosis and degradation by LSEC, a scavenging assay using radiolabeled formaldehyde treated serum albumin (^125^I-FSA) was performed. Briefly, LSEC were seeded on fibronectin coated 48-well plate at a cell density of 2.5 × 10^5^ cells/cm^2^. The cells were challenged with matched concentrations of clozapine or norclozapine (0-100-1000-10 000-25 000-50 000-75 000-100 000 nmol/L) for 30 min in serum free RPMI before a 2-hour incubation period with 20 000 cpm of ^125^I-FSA supplemented with 1% human serum albumin (Alburex, CSL Behring, Marburg, Germany). The assay was performed as previously described in Mao et al. [31] and Kruse et al. [32], and the radioactivity was measured using a Cobra II, Auto-Gamma detector (Packard Instruments, Laborel, Oslo, Norway). The degraded and cell associated fractions were then analyzed.

#### Statistical analysis

Statistical analysis was carried out using GraphPad Prism (version 10.6.1). Cell porosity scores were averaged for each mouse per treatment group prior to significance testing. The normality of distribution was assessed by Shapiro-Wilk test and evaluated using the quantile-quantile plots of the means, showing no deviation from normality. Differences in mean porosity among treatment groups were analysed using one-way analysis of variance (ANOVA), and Dunnett’s multiple comparison test was then applied to compare each treatment group with the corresponding untreated control. A significance threshold of *P* < 0.05 was used.

Dose-response relationships were analysed in R (version 4.5.2), using a segmented model [33], by first averaging porosity per concentration and fitting a linear model to test for a monotonic trend, then fitting segmented regression with up to the user selected maximum number of breakpoints. The best breakpoint count was chosen by Akaike information criterion (AIC), and an overall change in slope was evaluated by the Davies test. A significance threshold of *P* < 0.05 was used.

The results from the viability study were normalized to the corresponding untreated control group for each animal. Technical replicates were averaged for each mouse per treatment group, prior to performing statistical analysis (GraphPad Prism version 10.6.1). The normality of data distribution was assessed using the Shapiro-Wilk test and evaluated using the quantile-quantile plots of the means, showing no deviations from normality. One-way ANOVA was used to assess differences among treatment groups, and Dunnett’s multiple comparisons test was applied to identify the significant group differences. A significance threshold of *P* < 0.05 was used.

For the endocytosis assay, the degraded and the cell associated fraction were normalized to the corresponding untreated control group for each treatment group and each animal. Technical replicates were averaged prior to normalization. Ordinary one-way ANOVA was used to assess the differences among clozapine-challenged groups, followed by Dunnett’s multiple comparisons test. The statistical testing was carried out separately for the degraded fraction, cell associated fraction, and total endocytosis. For the norclozapine-challenged groups, Brown-Forsythe and Welch ANOVA was used when assessing the differences among the groups, as equal standard deviation could not be assumed, followed by Dunnett T3 multiple comparisons test. A significance threshold of *P* < 0.05 was used.

### Role of funders

The funders had no role in the planning or execution of this study.

## Results

### Patient data

TDM analyses of clozapine were available from 4 073 patients. 2 869 patients had available samples that were drawn within ten to 30 h after the last dose, of which 2 634 had available samples indicating monotherapy. Of these, samples from 89 patients had simultaneously been analysed for at least one MP (Supplementary Figure 1). Two included TDM measurements of clozapine and four of norclozapine were below the lower limit of quantification. One included TDM measurement had a clozapine and norclozapine concentration above the upper limit of quantification. No metabolic parameters were below the lower limit of quantification or above the upper limit of quantification. An overview of the included patients can be found in Table 1.

**Table 1:** Characteristics of the patients included in each analysis group.

	N	Mean age (range)	Sex (M/F)	Smoking (Y/N/unknown)
Triglycerides	57	42.0 (22–77)	33/24	6/11/40
LDL cholesterol	46	42.3 (23–77)	24/22	5/10/31
HDL cholesterol	59	42.1 (22–77)	33/26	7/11/41
Glucose	79	42.3 (20–84)	47/32	9/19/51
HbA1c	53	46.3 (22–84)	27/26	5/13/35

Some patients are included in two or more groups. Sex was recorded in the laboratory information management system at the time of blood sampling based on the national identity number.

**Fig. 1:**
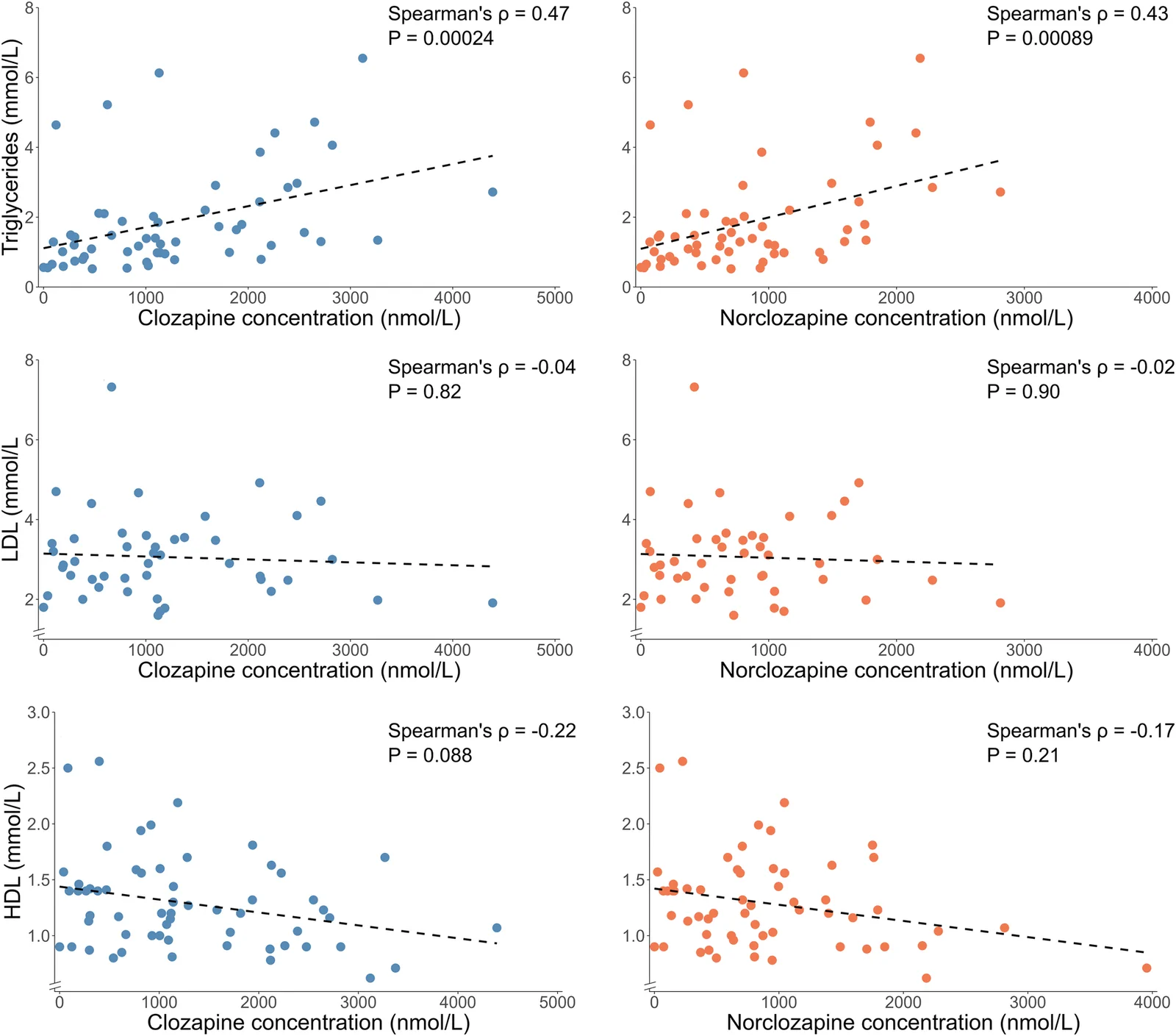
Correlations between clozapine/norclozapine serum concentrations and lipids. Observational data of clozapine and norclozapine serum concentrations, and their relationship to biochemical markers that were analysed in the same sample. The ρ and P values from Spearman’s rank correlation analysis are presented for each comparison. The dashed lines are for visualization purposes and are based on simple linear regression models.

**Fig. 2:**
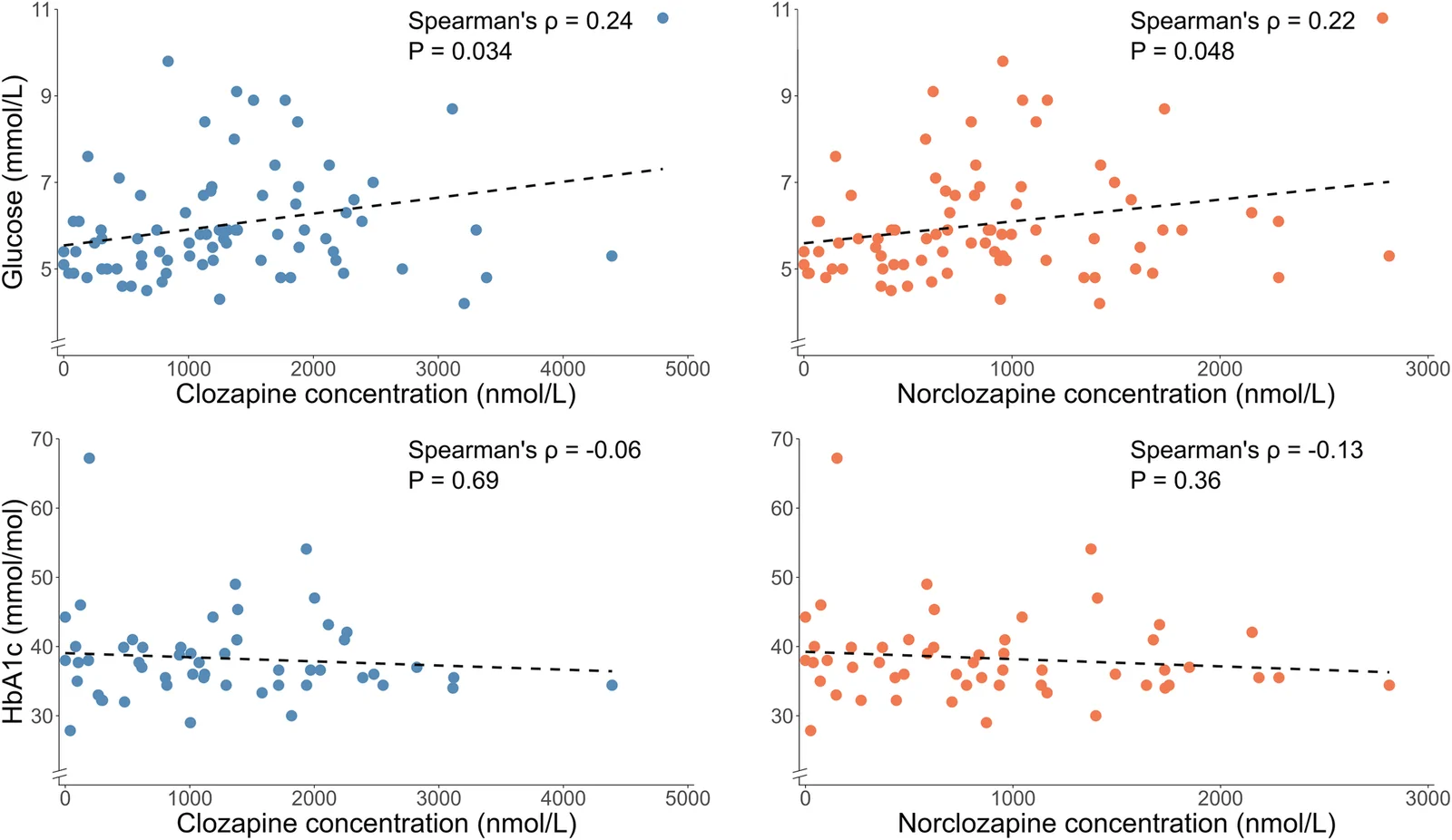
Correlations between clozapine/norclozapine serum concentrations and glucose/HbA1c levels. The ρ and P values from Spearman’s rank correlation analysis are presented for each comparison. The dashed lines are for visualization purposes and are based on simple linear regression models.

When adjusting for age, sex, and treatment duration, significantly positive correlations of clozapine concentration with triglycerides and glucose were found both by Spearman’s rank correlation analysis (Figs. 1 and 2) and by linear regression (Table 2 and Supplementary Table 2). No significant correlations of clozapine or norclozapine with LDL cholesterol, HDL cholesterol, or HbA1c were found.

**Table 2:** Simple linear regression (unadjusted) and multiple linear regression (adjusted for age, sex, and treatment duration) results.

Metabolic parameter	Clozapine					
	Unadjusted			Adjusted for age, sex and treatment duration		
	β coefficient	95% CI	R^2^	β coefficient	95% CI	Adj. R^2^
Triglycerides	0.60**	(0.24, 0.96)	0.17	0.64***	(0.28, 1.01)	0.25
LDL cholesterol	−0.07	(−0.41, 0.27)	0.004	−0.006	(−0.37, 0.35)	0.02
HDL cholesterol	−0.12*	(−0.22, −0.01)	0.08	−0.08	(−0.18, 0.02)	0.22
Glucose	0.37*	(0.07, 0.66)	0.07	0.33*	(0.02, 0.64)	0.08
HbA1c	−0.60	(−2.44, 1.26)	0.01	−0.13	(−2.13, 1.86)	−0.03
Triglyceride/HDL ratio	0.76**	(0.24, 1.27)	0.14	0.82**	(0.30, 1.33)	0.24

The specified metabolic parameter was used as the dependent variable, while the clozapine concentration was used as the independent variable. In the multiple regression analysis, age, sex, and treatment duration were used as independent variables in addition to the clozapine concentration. β coefficients of the clozapine concentrations only are presented, as these are the variables of interest in this study. The coefficients represent the change in metabolic parameter per 1000 nmol/L change in clozapine concentration. *, **, ***: *P* < 0.05, 0.01 and 0.001, respectively. Statistically significant β coefficients are written in bold.

To be able to compare the concentrations LSEC in patients are exposed to, to concentrations used in the in vitro experiments, maximum hepatic inlet concentrations were calculated. In patients, the clozapine doses used ranged from 12.5 mg to 800 mg, with a median of 300 mg (see Supplementary Table 4 for details). The maximum hepatic inlet concentration in each patient was calculated based on dose and measured systemic serum concentrations (Table 3) [22]. The median calculated maximum hepatic inlet concentration of unbound clozapine was 826 nmol/L (corresponding to a dose of 300 mg and a total systemic clozapine concentration (bound and unbound) of 622 nmol/L), with maximal concentrations reaching 2 211 nmol/L (corresponding to a dose of 800 mg and a total systemic clozapine concentration (bound and unbound) of 2 226 nmol/L). The norclozapine values in the table represent measured systemic serum concentrations, as the immediate contribution from a newly ingested clozapine dose to the hepatic inlet concentration of norclozapine will be negligible due to the time required for the drug to be metabolized.

**Table 3:** Distribution of maximum hepatic inlet concentrations in nmol/L.

	Min	25th percentile	Median	Mean	75th percentile	Max
Total (protein bound and unbound)						
Clozapine	541	9 493	13 773	13 969	18 563	36 854
Norclozapine	0	424	831	925	1 302	3955
Unbound						
Clozapine	32	570	826	838	1 114	2 211
Norclozapine	0	42.4	83.1	92.5	130.2	395.5

Clozapine concentrations are based on dose and measured systemic serum concentrations, calculated as described in the Methods section. Norclozapine concentrations represent measured systemic serum concentrations.

### In vitro studies

#### LSEC ultrastructure determined with scanning electron microscopy

**Fig. 3:**
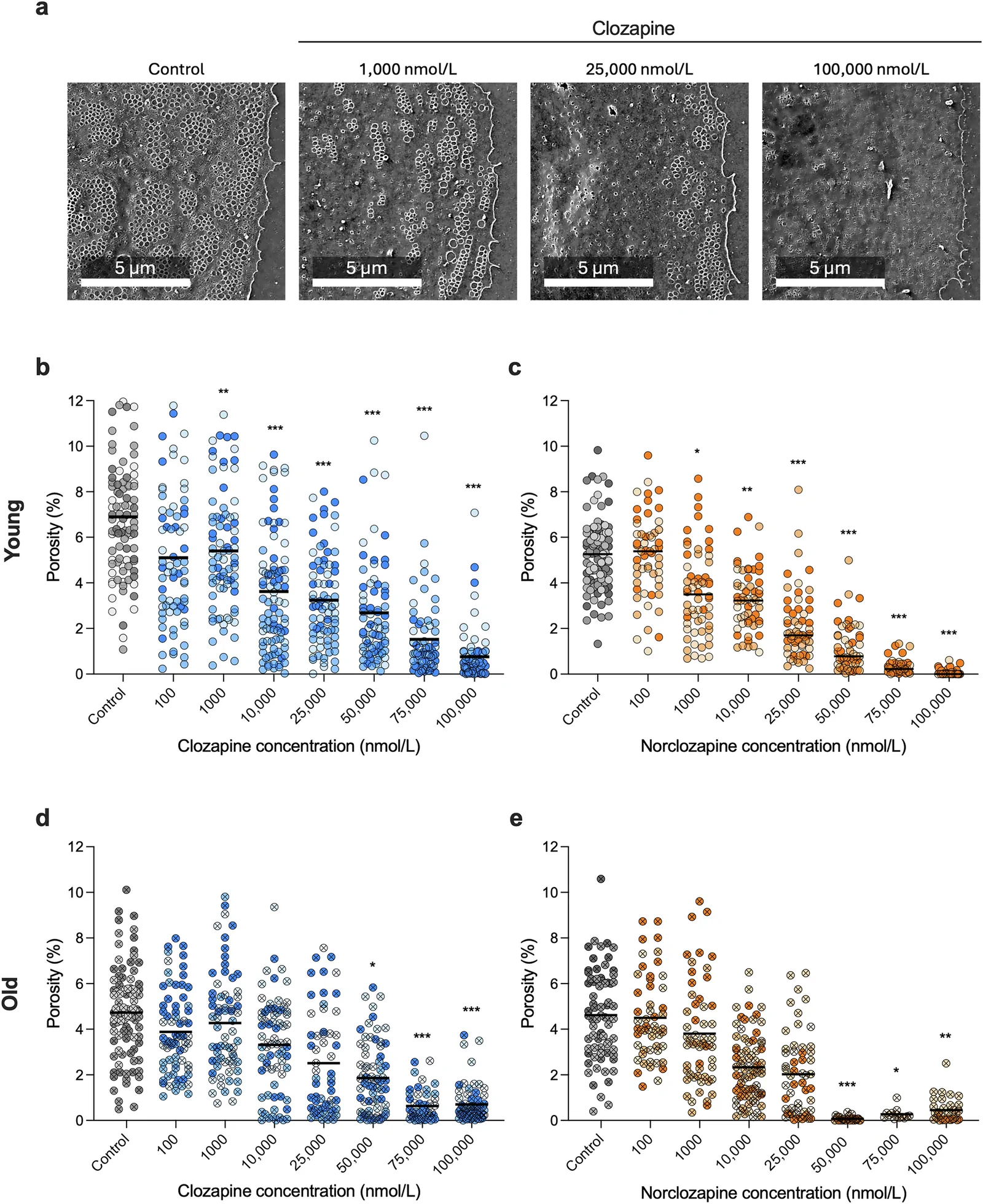
Effect of clozapine/norclozapine on LSEC porosity. Representative, high magnification SEM images of untreated LSEC, and LSEC treated with clozapine, isolated from young mice (**a**). The raw images were denoised using the Noise2Void-based method [48] to suppress acquisition noise, and contrast was adjusted by applying a linear stretch to the full 8-bit dynamic range, without introducing, removing or repositioning image features. No features were enhanced, obscured, moved, removed or added at any stage. Porosity of clozapine (**b,d**) and norclozapine (**c,e**) treated LSEC, freshly isolated from young (**b,c**) and old (**d,e**) mice. The cells were treated for 1 h, fixed, examined by electron microscopy, and fenestrations were scored. The graphs present the combined data from 3–4 animals, where each biological replicate is represented by a different shade of blue or orange, and each dot represents one cell. The mean is shown as a line. 15–20 cells were analysed per treatment group per animal. Ordinary one-way ANOVA, and Dunnett’s multiple comparison test was used for significance testing. *, **, ***: *P* < 0.05, < 0.005, < 0.001, respectively.

To evaluate the effect of clozapine and norclozapine on LSEC porosity, cells isolated from young and old mice were treated with matched concentrations of either clozapine or norclozapine for 1 h, fixed, and later examined by scanning electron microscopy. The images were quantitatively analysed to calculate fenestration diameter (Supplementary Table 6), LSEC porosity (Fig. 3), and fenestration frequency (Supplementary Figure 2). Typical examples of LSEC isolated from young mice, untreated, and treated with 1000, 25 000 or 100 000 nmol/L of clozapine, are shown in Fig. 3a. Treatment with either clozapine or norclozapine resulted in a dose-dependent reduction in LSEC porosity (Fig. 3). In young mice, a significant trend was observed across the concentrations for both compounds (P for trend = 0.000355 for clozapine and *P* = 0.00177 for norclozapine) (Supplementary Figure 3). LSEC exposed to concentrations of 1000 nmol/L and above of either clozapine or norclozapine showed a significant reduction in porosity, in comparison with the untreated control group. A similar dose-dependent decrease in LSEC porosity was observed in cells isolated from old mice (Fig. 3d, e), where a significant trend was detected for both compounds (P for trend = 0.000332 for clozapine and = 0.000328 for norclozapine) (Supplementary Figure 4). Treatment with concentrations of 50 000 nmol/L and above of either clozapine or norclozapine resulted in a significant reduction in LSEC porosity, when compared to untreated controls. A summary of the segmented linear regression can be found in Supplementary Table 7.

#### LSEC viability study

**Fig. 4:**
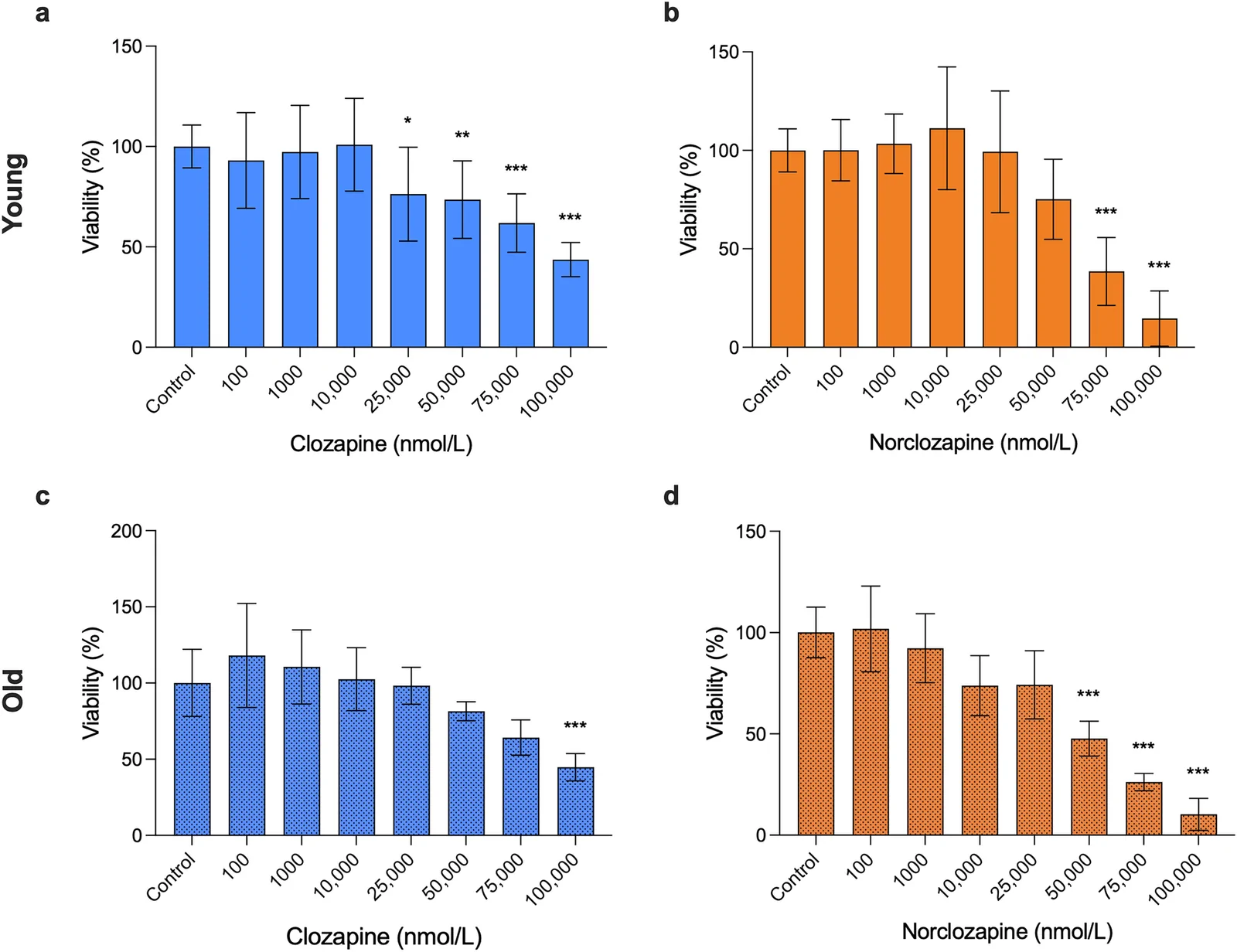
Effect of clozapine/norclozapine on LSEC viability. Resazurin viability assay performed on clozapine (**a,c**) and norclozapine (**b,d**) treated LSEC, freshly isolated from young (**a,** **b**) and old (**c,** **d**) mice. LSEC were pre-treated with clozapine or norclozapine 30 min prior to addition of 10% resazurin and further incubation for 3 h. The graphs (**a,** **b**) show the mean (±standard deviation) of the combined data from 9 and 4 young animals for clozapine and norclozapine respectively, and (**c,** **d**) the mean of combined data from 4 and 3 aged animals for clozapine and norclozapine respectively. Ordinary one-way ANOVA, and Dunnett’s multiple comparison test was used for significance testing. *, **, ***: *P* < 0.05, < 0.005, < 0.001, respectively.

To assess the potential toxicity of clozapine and norclozapine on LSEC isolated from young and old mice, resazurin assay was performed. LSEC were treated with matched concentrations of clozapine or norclozapine (Fig. 4). In cells isolated from young mice, a significant decrease in cell viability was observed after treatment with clozapine at concentrations above 25 000 nmol/L of clozapine, and with norclozapine at concentrations above 75 000 nmol/L (Fig. 4a, b). In LSEC isolated from old mice, the viability was significantly reduced at 100 000 nmol/L for clozapine, and above 50 000 nmol/L for norclozapine (Fig. 4c, d). No change in viability was detected at clinically relevant concentrations.

#### Endocytosis assay

**Fig. 5:**
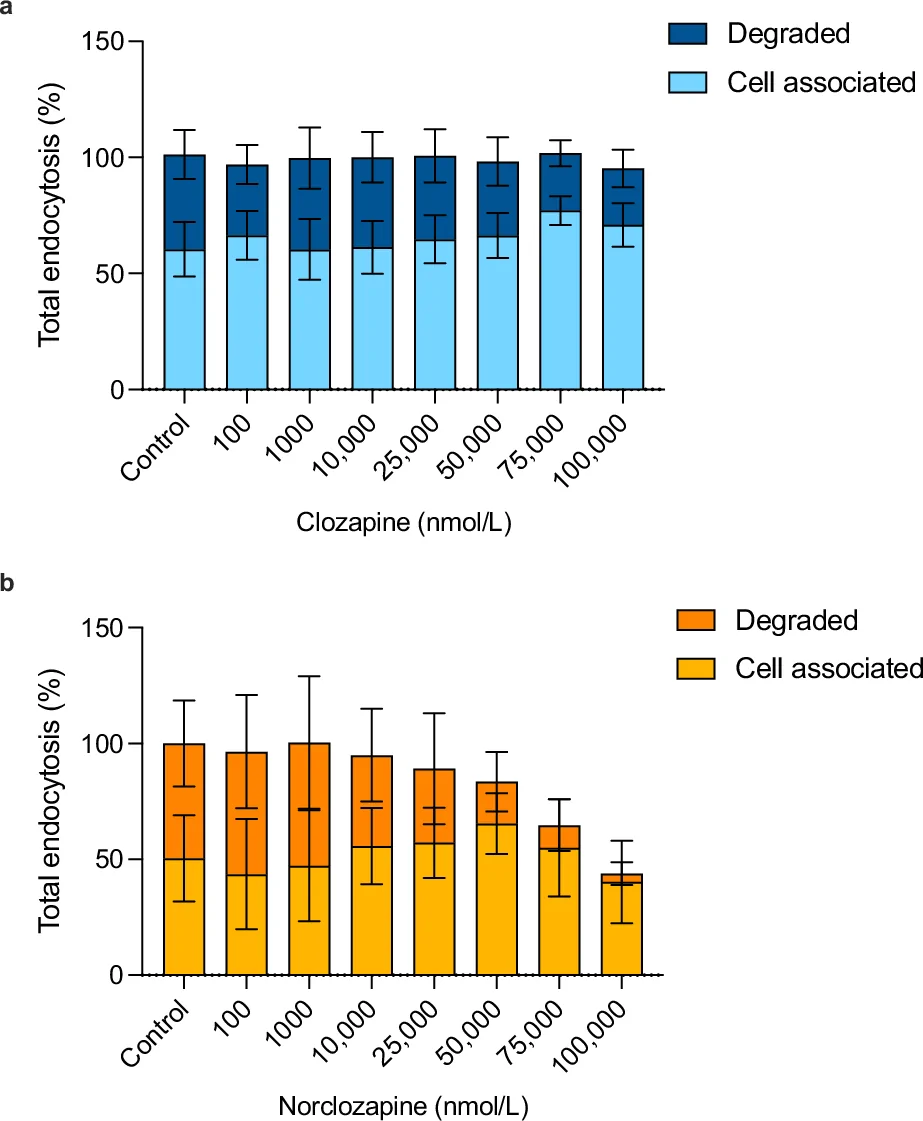
Effect of clozapine/norclozapine on LSEC endocytosis. Endocytosis assay was performed using ^125^I radiolabeled formaldehyde treated serum albumin for quantitative studies of endocytosis in LSEC freshly isolated from young mice, after a 30 min challenge with clozapine (**a**) or norclozapine (**b**). The graphs show mean (±standard deviation) of the combined data from 3–5 animals for clozapine, and 3–4 animals for norclozapine. Ordinary one-way ANOVA, and Brown-Forsythe and Welch ANOVA were used for significance testing of clozapine and norclozapine challenged groups respectively, followed by Dunnett’s and Dunnett T3 multiple comparisons tests. When assessing total endocytosis, *P* = 0.0514 for norclozapine 100 000 nmol/L, and *P* = 0.0651 when assessing the degraded fraction.

To assess the functionality of LSEC after challenge with either clozapine or norclozapine, a scavenging assay was performed to quantify endocytosis and the degradation of radiolabeled ^125^I-FSA by LSEC. Neither clozapine nor norclozapine resulted in a statistically significant reduction in total endocytosis by LSEC (Fig. 5a, b). There was no significant difference in either the degraded fractions or the cell associated fractions in any of the treatment groups for either clozapine (Fig. 5a) or norclozapine (Fig. 5b) when compared to the untreated control group.

## Discussion

Our study provides potentially critical insight into the pathophysiological effects of clozapine and its main metabolite norclozapine, associated with adverse metabolic effects during treatment. Through our findings of impaired LSEC function, we have uncovered a potential mechanism with a direct relevance to clinical practice. We hypothesized that hypertriglyceridemia in clozapine-treated patients may result from decreased hepatic clearance of lipids caused by defenestration of LSEC. Our observations that serum concentrations of clozapine and norclozapine correlate positively with triglyceride levels in patients, in concert with a significant reduction of in vitro LSEC porosity caused by similar concentrations, are consistent with this hypothesis. With this, we propose a potential treatment target for weight gain-independent dyslipidaemia in clozapine treated patients.

Elevated triglyceride and cholesterol levels among clozapine users compared to other antipsychotic drugs or placebo are well documented [5]. Still, the dose-dependency of this metabolic effect was unclear, as previous studies investigating the relation between lipid levels and clozapine serum concentration are small and show conflicting results [34, 35]. In the present study, which includes a high number of patients with clozapine serum samples that are time-matched to metabolic parameters, we found that increasing clozapine and norclozapine levels correlated with increasing triglyceride levels, even after adjusting for age and gender. In contrast, no correlation was found between clozapine or norclozapine and HDL or LDL levels. This implies the existence of a distinct mechanism that specifically affects triglyceride metabolism and that becomes increasingly prominent with increasing clozapine concentrations within the therapeutic range.

The in vitro studies provide a biologically plausible mechanistic explanation for our observations in the patient data; we show that both clozapine and norclozapine cause a concentration-dependent reduction in porosity in freshly isolated LSEC. The change in porosity is primarily caused by the decrease in the number of fenestrations, but not their size. Interestingly, this trend is analogous in cells isolated from both young and old mice, suggesting that the effect is age independent.

The reduction in porosity was observed at 1000 nmol/L for clozapine and norclozapine in LSEC isolated from young mice. The effective clozapine concentrations overlap with the calculated concentration of clozapine that enters the liver after drug-intake, namely the hepatic inlet concentrations of unbound drug, in included clozapine-treated individuals. Importantly, LSEC viability was not affected at these concentrations, suggesting that clozapine and norclozapine induce alterations in LSEC porosity at clinically relevant concentrations without compromising cell viability. At supra-therapeutic concentrations (50 000–100 000 nmol/L), the viability was reduced by 20–50% compared to the untreated control, indicating a toxic effect at higher concentrations. In LSEC isolated from aged mice, norclozapine induced a more profound defenestration and reduction in viability at supra-therapeutic concentrations than what is seen in cells derived from young mice, indicating that ageing increases the susceptibility to drug-induced toxicity, e.g. after overdose.

Under physiological conditions, lipoproteins must be transferred from the bloodstream in the sinusoidal lumen and across LSEC fenestrations into the space of Disse before processing by hepatocytes [36]. When LSEC porosity is reduced, the transport of larger triglyceride-rich chylomicrons to the liver will increasingly be inhibited, leading to hypertriglyceridemia and ultimately atherosclerosis. At the same time, smaller lipoproteins such as LDL and HDL cholesterol will still be able to flow freely. Thus, a reduction in LSEC porosity is a potential explanation for the concentration-dependent increase only in triglycerides, as observed in our patient data. As discussed below, LSEC defenestration is also associated with hyperglycaemia; this, in turn, stimulates lipogenesis and further contributes to elevated triglyceride levels [37]. The observed effect of clozapine – in serum samples from clozapine-treated patients, as well as directly on isolated LSEC – is analogous to the atherogenic effects of ageing, where increasing triglyceride levels have been associated with decreasing LSEC porosity [17, 20]. Age-related LSEC defenestration is now recognized as a risk factor for post-prandial hyperlipidaemia [38] and insulin resistance [21]. In the present study, our findings suggest that clozapine exposure can induce an old-age LSEC phenotype associated with dyslipidaemia.

While the primary target in the prophylactic treatment of patients with dyslipidaemia is lowering LDL cholesterol, triglyceride-rich chylomicron remnants are also important in the development of atherosclerosis [39]. Notably, the ratio between triglycerides and HDL has been shown to correlate strongly with risk of cardiovascular disease [40, 41]. We show that the triglyceride/HDL ratio increased with both increasing concentrations of clozapine and norclozapine in patient samples, as expected considering their negative and dose-dependent effect on LSEC porosity in vitro.

Further, the patient data shows a correlation between blood glucose levels and both clozapine and norclozapine serum concentrations. This reinforces our hypothesis of LSEC being involved in the negative metabolic effects of clozapine treatment, as reduced LSEC-porosity is associated with insulin resistance [17, 21]. The same relationship, however, is not seen for HbA1c in our patient data. This is surprising as HbA1c reflects the mean blood glucose levels over the weeks preceding blood sampling. A possible explanation for these contradictory results is that a selection bias may exist regarding which clozapine users are being tested for HbA1c, as more patients are included with blood glucose levels (N = 79) than HbA1c levels (N = 53). The patients being tested for HbA1c are more likely to have previously shown glucose abnormalities, and appropriate treatment could have already been started, considering only the last available sample for each patient is included in the analysis. Such concurrent treatment is a likely confounder in evaluating a relation between clozapine serum concentrations and HbA1c, as it will affect glucose metabolism and also could refenestrate LSEC, as established for the antidiabetic metformin [42].

Although it is established that LSEC defenestration is detrimental for health, the exact mechanism causing this is still not yet fully understood. However, cytoskeletal rearrangement appears to be the main contributor to the defenestration process [43]. Fenestrations are known to be dynamic structures and are responsive to a range of exogenous compounds. In fact, several compounds have been shown to increase the number or diameter of fenestrations in isolated LSEC from young and old animals, including sildenafil [44]. In murine models of non-alcoholic steatohepatitis, re-fenestration was associated with recovery [43, 45]. Zapotoczny et al. [43] also demonstrated that defenestration can be reversible under certain conditions, as cytochalasin B induced fenestrations in de-fenestrated cells in vitro. Although defenestration is regarded as a hallmark of ageing and associated with certain liver disorders, studies suggest that these changes may not be permanent, offering potential avenues for therapeutic intervention. In our recent study [46], we showed that targeted delivery of sildenafil using poly(alkyl-cyanoacrylate) (PACA) nanoparticles re-fenestrated poorly fenestrated LSEC isolated from old mice. This nanoparticle-based treatment method has the potential to alleviate the severe, weight-independent metabolic effects of clozapine.

### Strengths and limitations

A strength of this study is that the suggested mechanism for clozapine induced hypertriglyceridemia has been investigated both experimentally in vitro and observationally in patient samples from a real-world clinical setting. The patient data were selected based on stringent inclusion and exclusion criteria, resulting in a high confidence in the validity of the data. Inclusion of only the last available sample for each patient ensured that the metabolic effects of long-term treatment were incorporated, as opposed to most prospective studies, where follow-up times are short.

A limitation of the patient data is that the smoking status was not stated on the requisition for a large number of patients. Smoking increases the metabolism of clozapine by inducing CYP1A2, and smoking status could therefore affect the results by simultaneously decreasing the clozapine concentration and increasing triglyceride levels. However, this dynamic would suppress the association between clozapine concentration and triglycerides rather than act as a confounder. Nicotine by itself also causes LSEC defenestration, further leading to suppression of the association rather than confounding it [17]. Additionally, patients are normally closely followed with TDM and clinical evaluation, leading to doses being adjusted to account for smoking status. Information about BMI, co-prescribed medication, and whether blood was drawn fasting or not was unfortunately not available. In addition, data were not available on lipid-lowering therapy, antidiabetic treatment, diet or other metabolic comorbidities. These factors may influence triglycerides, glucose, and potentially LSEC phenotype and would have improved the analysis.

As the TDM laboratory from which the data were collected is highly specialized and receives samples from across the country, a large number of TDM samples were available for inclusion. Information on MP results was only available for samples analysed at the biochemical laboratory in the same hospital, which primarily serves patients living within the hospital’s area of responsibility, resulting in a much smaller number of patients with available data on both TDM and MPs. A selection bias might still be present in the patient data, as requesting the analysis of both TDM and MPs is more likely for patients with a high metabolic risk profile. Such a bias has minimal effect on the results of this study, as the correlation between TDM and MPs is expected to be similar among those with high metabolic risk compared to those with low metabolic risk.

The hepatic inlet concentrations are model-based estimates that rely on population assumptions, and they do not represent measured sinusoidal concentrations in individual patients. Conversely, the nominal concentrations added to serum-free culture medium do not necessarily equal the unbound concentration at the LSEC membrane. The overlap between estimated patient exposure and effective in vitro concentrations should therefore be interpreted as supportive of clinical plausibility, not as evidence of equivalent exposure.

A potential limitation of the in vitro studies is that murine LSEC were used, and these cells may not respond to exogenous substances in the same way as human LSEC. Although the concentrations required for defenestration might differ between humans and mice, the fact that the murine studies showed a concentration-dependent effect increases the likelihood that a similar concentration-dependent effect is also present in humans. Additionally, the treatment was acute (one hour), while patients would have chronic exposure to repeated doses of clozapine. Moreover, while no albumin was added to the cell cultures, the free fraction of clozapine and norclozapine exposing the cells can be lower than the added concentration due to binding to other components in the media [47].

## Conclusion

Overall, these findings are consistent with a plausible LSEC-mediated mechanism linking clozapine exposure to hypertriglyceridemia. Further validation in in vivo models and prospective clinical studies is required to determine whether clozapine-induced LSEC defenestration contributes causally to metabolic adverse effects in patients and whether this pathway can be therapeutically targeted. Ultimately, this could alleviate the substantial burden of schizophrenia, which remains one of the most costly and debilitating disorders worldwide.

## Supplementary information

Supplements

## Data Availability

The observational data from routine patient samples will not be made publicly available due to patient privacy concerns and local regulations. The experimental data set will be made available upon reasonable request to the corresponding author.
