# Supplementary material for: Association between clozapine-induced hypertriglyceridemia and defenestration of liver sinusoidal endothelial cells
Source: Transl Psychiatry. 2026 Aug 6;16:577. doi: 10.1038/s41398-026-04328-4 (PMC13683405; doi:10.1038/s41398-026-04328-4)
Supplement: Supplementary file 1 [file 41398_2026_4328_MOESM1_ESM.docx]

**SUPPLEMENTS**

**Supplementary Figure 1: Patient inclusion and exclusion**

The bottom row represents the number of patients with available samples analysed for both the metabolic parameter and clozapine serum concentration.

### Supplementary Figure 2: Effect of clozapine/norclozapine on fenestration frequency in LSEC

Fenestration frequency (number of fenestrations per cell area) of clozapine (a,c) and norclozapine (b,d) treated LSEC, freshly isolated from young (a,b) and old (c,d) mice. The cells were treated for 1 hour, fixed, examined by electron microscopy, and fenestrations were scored. The graphs present the combined data from 3–4 animals, where each biological replicate is represented by a different shade of blue or orange, and each dot represents one cell. The mean is shown as a line. 15–20 cells were analysed per treatment group per animal. One-way analysis of variance (ANOVA), and Dunnett’s multiple comparison test was used for significance testing.

*, **, ***: P < 0·05, < 0·005, < 0·001, respectively.

Supplementary Figure 3: Dose-response quality control curves for young mice
Dose-response quality control curves for clozapine (A) and norclozapine (B) treated LSEC from young mice, showing the mean ± SD ribbon. A linear model revealed a trend across concentrations. Dotted line represents the breakup point chosen by AIC. Details are summarized in Supplementary Table 7. Plot generated using ggplot2 as described by Wickham (2016) (1).

Supplementary Figure 4: Dose-response quality control curves for old mice
Dose-response quality control curves for clozapine and norclozapine treated LSEC from old mice, showing the mean ± SD ribbon. A linear model revealed a trend across concentrations. Dotted line represents the breakup point chosen by AIC. Details are summarized in Supplementary Table 7. Plot generated using ggplot2 as described by Wickham (2016) (1).

Supplementary Figure 5: Exploratory analysis of triglyceride/clozapine ratio vs age
A significant positive correlation, as determined by simple linear regression (p < 0.05), is observed. This may indicate an increasing vulnerability to clozapine-induced hypertriglyceridemia in older patients. However, the correlation becomes non-significant when one outlier (encircled in red) is removed. Spearman’s rank correlation coefficient is non-significant both with and without the inclusion of the outlier. Overall, the results from this analysis should be interpreted with caution.

Supplementary Figure 6: Exploratory analysis of triglyceride/norclozapine ratio vs age
A significant positive correlation, as determined by simple linear regression (p < 0.05), is observed. This may indicate an increasing vulnerability to norclozapine-induced hypertriglyceridemia in older patients. However, the correlation becomes non-significant when one outlier (encircled in red) is removed. Spearman’s rank correlation coefficient is non-significant both with and without the inclusion of the outlier. Overall, the results from this analysis should be interpreted with caution.

| **Parameter** | **Assumption** | **Comment** | **Impact** |
| --- | --- | --- | --- |
| fu_p_ | 0.06 | Low variability under normal circumstances, coefficient of variation of 19% according to Schaber et al. Can decrease significantly during severe inflammation. | Low |
| F_a_xF_g_ | 0.95 | 0.90 to 0.95 according to the SPC, not affected by food intake. Whether 0.90 or 0.95 is used, has low impact on final concentration. | Low |
| k_a_ | 1.3 | Jerling et al. states the median with 25% and 75% quantiles to be 1.30 (0.87, 1.72) for men and 1.24 (0.80, 1.50) for women. These ranges translates to over two-fold variation in maximum hepatic inlet concentrations. | High |
| Q_H_ | 97 L/h | Standard value for a 70-kg person used by The European Medicines Agency (EMA), the US Food and Drug Administration (FDA), and the Japanese Pharmaceutical and Medical Devices Agency (PMDA) according to Parkinson. This value changes proportionally with weight. | Moderate |
| R_b_ | 0.9 | Might be affected by haematocrit and concentration of binding proteins according to Flanagan et al. | Moderate |

**Supplementary Table 1. Assumptions and sensitivity table for hepatic inlet concentration calculations.**

| Metabolic parameter | **Norclozapine** | | | | | | |  | |  |
| --- | --- | --- | --- | --- | --- | --- | --- | --- | --- | --- |
|  | Unadjusted | | |  | Adjusted for age, sex and treatment duration | | | |  | |
|  | β coefficient | 95% CI | R^2^ |  | β coefficient | 95% CI | Adj.  R^2^ | |  | |
| Triglycerides | **0.90**** | (0.37, 1.43) | 0.17 |  | **0.96***** | (0.43, 1.48) | 0.26 | |  | |
| LDL cholesterol | -0.09 | (-0.61, 0.42) | 0.003 |  | 0.03 | (-0.54, 0.59) | -0.02 | |  | |
| HDL cholesterol | **-0.14*** | (-0.28, -0.01) | 0.07 |  | -0.11 | (-0.24, 0.03) | 0.22 | |  | |
| Glucose | **0.50*** | (0.04, 0.97) | 0.06 |  | 0.43 | (-0.06, 0.92) | 0.06 | |  | |
| HbA1c | -1.05 | (-3.63, 1.53) | 0.01 |  | -0.36 | (-3.15, 2.43) | -0.02 | |  | |
| Triglyceride/  HDL ratio | **1.15**** | (0.39, 1.91) | 0.15 |  | **1.26**** | (0.50, 2.01) | 0.25 | |  | |

**Supplementary Table 2. Association between norclozapine concentration and metabolic parameters.**

Simple linear regression (unadjusted) and multiple linear regression (adjusted for age, sex and treatment duration) results. The specified metabolic parameter was used as the dependent variable, while the norclozapine concentration was used as the independent variable. In the multiple regression analysis, age, sex, and treatment duration, were used as independent variables in addition to the norclozapine concentration. β coefficients of the norclozapine concentrations only are presented, as these are the variables of interest in this study. The coefficients represent the change in metabolic parameter per 1000 nmol/L change in norclozapine concentration. *, **, ***: P < 0.05, 0.01 and 0.001, respectively. Statistically significant β coefficients are written in bold.

| Metabolic parameter | **Clozapine** | | | | | | |  |
| --- | --- | --- | --- | --- | --- | --- | --- | --- |
|  | Unadjusted | | |  | Adjusted for age, sex and treatment duration | | | |
|  | β coefficient | 95% CI | R^2^ |  | β coefficient | 95% CI | Adj.  R^2^ | |
| Triglycerides | **0.46**** | (0.10, 0.82) | 0.28 |  | **0.52**** | (0.18, 0.87) | 0.27 | |
| LDL cholesterol | -0.04 | (-0.36, 0.29) | 0.002 |  | 0.01 | (-0.31, 0.34) | -0.02 | |
| HDL cholesterol | -0.09 | (-0.18, 0.002) | 0.06 |  | -0.08 | (-0.16, 0.0004) | 0.17 | |
| Glucose | 0.14 | (-0.16, 0.45) | 0.02 |  | 0.15 | (-0.29, 0.59) | 0.04 | |
| HbA1c | -0.37 | (-1.52, 0.78) | 0.01 |  | 0.79 | (-0.54, 2.13) | 0.25 | |
| Triglyceride/  HDL ratio | 0.43 | (-0.02, 0.87) | 0.21 |  | **0.53*** | (0.02, 1.03) | 0.22 | |
|  | **Norclozapine** | | | | | | | |
|  | Unadjusted | | |  | Adjusted for age, sex and treatment duration | | | |
|  | β coefficient | 95% CI | R^2^ |  | β coefficient | 95% CI | Adj.  R^2^ | |
| Triglycerides | **0.72**** | (0.28, 1.16) | 0.30 |  | **0.79***** | (0.37, 1.21) | 0.28 | |
| LDL cholesterol | -0.03 | (-0.52, 0.47) | 0.003 |  | 0.08 | (-0.49, 0.65) | -0.01 | |
| HDL cholesterol | **-0.11*** | (-0.21, -0.02) | 0.06 |  | **-0.10*** | (-0.19,  -0.01) | 0.17 | |
| Glucose | 0.16 | (-0.17, 0.49) | -0.002 |  | 0.09 | (-0.30, 0.49) | 0.02 | |
| HbA1c | -0.63 | (-2.24, 0.98) | 0.01 |  | 0.99 | (-0.78, 2.77) | 0.24 | |
| Triglyceride/  HDL ratio | **0.72*** | (0.07, 1.37) | 0.25 |  | **0.85**** | (0.26, 1.45) | 0.26 | |

**Supplementary Table 3. Robust regression using MM-estimation for the association between TDM clozapine/norclozapine concentrations and metabolic parameters**. The specified metabolic parameter was used as the dependent variable, while the clozapine/norclozapine concentration was used as the independent variable. In the multiple regression analysis, age, sex, and treatment duration, were used as independent variables in addition to the clozapine/norclozapine concentration. β coefficients of the clozapine/norclozapine concentrations only are presented, as these are the variables of interest in this study. The coefficients represent the change in metabolic parameter per 1000 nmol/L change in clozapine/norclozapine concentration. *, **, ***: P < 0.05, 0.01 and 0.001, respectively. Statistically significant β coefficients are written in bold.

|  | **Number of samples** | **Number of patients** | **Mean dose ± SD** | **Median dose**  **(25^th^ percentile –**  **75^th^ percentile)** |
| --- | --- | --- | --- | --- |
| **Samples with TDM** | 33 304 | 2 634 | 359 ± 197 | 350 (200 – 475) |
| **Samples with TDM and MP** | 280 | 89 | 393 ± 191 | 400 (250 – 500) |

Supplementary Table 4. Comparison of doses used in samples with and without metabolic parameters available
Clozapine doses (in mg) used in all samples where therapeutic drug monitoring (TDM) has been performed, compared to daily doses used in samples where both TDM and at least one metabolic parameter (MP) has been analyzed. The exclusion criteria were applied prior to analysis.

|  | **Number of samples** | **Number of patients** | **Mean concentration ± SD** | **Median concentration**  **(25^th^ percentile –**  **75^th^ percentile)** |
| --- | --- | --- | --- | --- |
| **Samples with TDM** | 33 304 | 2 634 | 1299 ± 815 | 1166 (698 – 1734) |
| **Samples with TDM and MP** | 280 | 89 | 1370 ± 995 | 1103 (564 – 2010) |

Supplementary Table 5. Comparison of clozapine concentrations in samples with and without metabolic parameters available
Clozapine concentrations (in nmol/L) in all samples where therapeutic drug monitoring (TDM) has been performed, compared to clozapine concentrations in samples where both TDM and at least one metabolic parameter (MP) has been analyzed. The exclusion criteria were applied prior to analysis.

| Average fenestration diameter (nm) ± SD |  | Concentration (nmol/L) | Control | 100 | 1000 | 10 000 | 25 000 | 50 000 | 75 000 | 100 000 |
| --- | --- | --- | --- | --- | --- | --- | --- | --- | --- | --- |
|  | **Young** | Clozapine | 161 ± 49 | 158 ± 46 | 159 ± 48 | 158 ± 49 | 163 ± 50 | 179 ± 56 | 175 ± 51 | 171 ± 55 |
|  |  | Norclozapine | 139 ± 42 | 135 ± 40 | 140 ± 39 | 139 ± 38 | 156 ± 46 | 163 ± 53 | 160 ± 63 | 176 ± 76 |
|  | **Old** | Clozapine | 165 ± 53 | 175 ± 62 | 167 ± 53 | 170 ± 56 | 176 ± 57 | 177 ± 57 | 183 ± 63 | 198 ± 66 |
|  |  | Norclozapine | 160 ± 55 | 164 ± 56 | 162 ± 55 | 172 ± 60 | 172 ± 60 | 173 ± 63 | 171 ± 65 | 149 ± 55 |

**Supplementary Table 6.** Fenestration size (diameter) in nm, expressed as an average of the 3-4 biological replicates with standard deviation.

| **Drug/age** | **Linear slope** | **Linear p** | **Linear r2** | **Segmented breakpoints** | **Segmented Breakpoint p** | **Segmented slopes** | **Segmented AIC*** |
| --- | --- | --- | --- | --- | --- | --- | --- |
| Clozapine  young | -0.05 | 0.000355 | 0.90 | 3.3156 | 0.0716 | -0.5719  -0.0400 | 16.17 |
| Norclozapine  young | -0.05 | 0.001770 | 0.83 | 1.0501  65.7648 | 0.0971 | -1.6483  -0.0506  0.0033 | 1.17 |
| Clozapine  old | -0.04 | 0.000332 | 0.90 | 0.1001  74.9999 | 0.1250 | -9.9603  -0.0423  0.0021 | 7.02 |
| Norclozapine  old | -0.04 | 0.003280 | 0.79 | 2.6462  49.9998 | 0.0433 | -0.5865  -0.0587  0.0009 | 5.11 |

Supplementary Table 7. Summary table of segmented linear regression
Dose response of porosity for clozapine and norclozapine treated LSEC isolated from young and old mice. Concentrations tested were 0, 100, 1000, 10 000, 25 000, 50 000, 75 000, 100 000 nmol/L, across 3–4 animals and 24 observations. The best breakpoint count was chosen by AIC, and overall change in slope was evaluated by the Davies test. *Akaike information criterion

| **Liver parameter** | **Spear-man’s ρ**  **(P value)** | **Clozapine** | | | | | | |  | |
| --- | --- | --- | --- | --- | --- | --- | --- | --- | --- | --- |
|  |  | Unadjusted | | |  | Adjusted for age, sex, and treatment duration | | | |  |
|  |  | β coefficient | 95% CI | R^2^ |  | β coefficient | 95% CI | Adj.  R^2^ | |  |
| ALT | 0.20 (0.060) | **5.17*** | (0.83, 9.50) | 0.06 |  | 4.01 | (-0.63, 8.65) | 0.05 | |  |
| AST | -0.03 (0.80) | 1.85 | (-1.27, 4.97) | 0.01 |  | 2.20 | (-1.21, 5.62) | -0.04 | |  |
| GT | 0.18  (0.11) | 7.34 | (-12.89, 27.57) | 0.01 |  | 10.51 | (-11.53, 32.56) | 0.02 | |  |
| ALP | 0.15  (0.22) | **10.33*** | (0.48, 20.18) | 0.06 |  | **14.00*** | (3.28, 24.72) | 0.06 | |  |
| Bilirubin | 0.00 (0.98) | -0.10 | (-3.75, 3.55) | 0.0001 |  | -0.85 | (-5.71, 4.00) | -0.05 | |  |
|  |  |  |  |  |  |  |  |  | |  |
|  |  | **Norclozapine** | | | | | | | |  |
|  |  | Unadjusted | | |  | Adjusted for age, sex, and treatment duration | | | |  |
|  |  | β coefficient | 95% CI | R^2^ |  | β coefficient | 95% CI | Adj.  R^2^ | |  |
| ALT | 0.22 (0.042) | **10.32**** | (3.71, 16.93) | 0.10 |  | **8.82*** | (1.62, 16.02) | 0.09 | |  |
| AST | -0.04 (0.79) | 2.92 | (-2.15, 7.98) | 0.24 |  | 3.62 | (-1.98, 9.21) | -0.04 | |  |
| GT | 0.29  (0.01) | 21.55 | (-10.40, 53.50) | 0.02 |  | 30.15 | (-5.21, 65.52) | -0.01 | |  |
| ALP | 0.18  (0.15) | **17.02*** | (2.13, 31.91) | 0.07 |  | **22.98**** | (6.61, 39.35) | 0.08 | |  |
| Bilirubin | 0.09  (0.68) | -0.87 | (-7.89, 6.16) | 0.003 |  | -2.93 | (-11.91, 6.06) | -0.03 | |  |

**Supplementary Table 8. Association between clozapine/norclozapine concentrations and liver parameters.** Simple linear regression (unadjusted) and multiple linear regression (adjusted for age, sex and treatment duration) results. The specified liver parameter was used as the dependent variable, while the clozapine/norclozapine concentration was used as the independent variable. In the multiple regression analysis, age, sex, and treatment duration, were used as independent variables in addition to the norclozapine concentration. β coefficients of the clozapine/norclozapine concentrations only are presented, as these are the variables of interest in this study. The coefficients represent the change in liver parameter per 1000 nmol/L change in clozapine/norclozapine concentration.
*, **, ***: P < 0.05, 0.01 and 0.001, respectively. Statistically significant β coefficients are written in bold.

### Reagents

Fibronectin was isolated from human plasma using Gelatin-Sepharose 4B (Cat. No. 17-0956-01, GE Healthcare, Sydney, Australia) according to the manufacturer’s instructions. The following reagents were obtained from Sigma-Aldrich, St. Louis, US/Mannheim, Germany: bovine serum albumin, heat shock fraction, pH 7, ≥98% (Cat. No. A7906); Liberase™ TM Research grade (Cat. No. 5401127001); sodium cacodylate trihydrate ≥98% (Cat. No. C025); RPMI1640 (Cat. No. R0883); Penicillin-Streptomycin (Cat. No. 4333) ethanol, pure, anhydrous ≥99.5% (Cat. No. 459836); hexamethyldisilazane (HMDS) reagent grade, ≥99% (Cat. No. 440191). For MACS cell isolation, the following reagents were obtained from Miltenyi Biotec, Bergisch Gladbach, Germany: CD-146 (LSEC) MicroBeads mouse (Cat. No. 130-092-007); autoMACS rinsing solution (Cat. No. 130-091-222); MACS BSA Stock Solution (Cat. No. 130-091-376); MS Columns (Cat. No. 130-042-201); pre-separation filters (70 µm) (Cat. No. 130-095-823). Osmium tetroxide 4%, aqueous solution (DG) (Cat. No. C011) was obtained from ProSciTech, Thuringowa, Australia. Paraformaldehyde 16% solution, EM Grade (Cat. No. 15710) was obtained from Electron Microscopy Sciences, Hatfield, USA. Glutaraldehyde 25% solution, EM Grade (Cat. No. 16210) was obtained from Laborimpex, Brussel, Belgium. CultureWell™ Removable Chambered Coverglass (Cat. No. CS16) was obtained from Grace Biolabs, Bend, Oregon, US. LDH-Glo™ Cytotoxicity Assay (Cat. No. J2380) was obtained from Promega, Madison, USA. alamarBlue™ Cell Viability Reagent (Cat. No. DAL1025) was obtained from Thermo Fisher Scientific, Eugene, USA. PACA reagents: 2-EBCA (Cuantum Medical Cosmetics, Barcelona, Spain), Miglyol 812 (IOI Oleo), Brij L23 (Cat. No. P1254, Sigma-Aldrich), Pluronic F-127 (Cat. No. P2443, Sigma-Aldrich), Kolliphor HS-15 (Cat. No. 4296, Sigma-Aldrich), docusate sodium (Cat. No. D-1685, Sigma-Aldrich), 3M HCl in methanol (Cat. No. 90964, Supelco). Clozapine (Cat. No. C6305) and N-Desmethylclozapine (Cat. D5676) were obtained from Sigma-Aldrich Chemie, Schnelldorf, Germany.

### R script for patient data selection

#Keep only adults

included <- included |>

filter(age > 17)

length(unique(included$pat_id))

#Add column indicating if time check is passed

included_mdiff <- included |>

mutate(mdiff_check = case_when(

mdiff < 600 ~ FALSE,

mdiff > 1800 ~ FALSE,

is.na(mdiff) ~ FALSE,

mdiff >= 600 & mdiff <=1800 ~ TRUE

))

length(unique(included_mdiff$pat_id))

#Keep serum concentration measurements only if time check is passed

included_mdiff <- included_mdiff |>

filter(mdiff_check == TRUE)

length(unique(included_mdiff$pat_id))

#Make a new data frame without metabolites, and keep only sample ids indicating monotherapy

monotherapy <- antipsychotics_all |>

filter(sub_code == "SRIS" | sub_code == "SOLA" | sub_code == "SLEM" | sub_code == "SPRF" | sub_code == "SQUE" | sub_code == "SKLP" | sub_code == "SHAL" | sub_code == "SZUK" | sub_code == "SAMI" | sub_code == "SARI" | sub_code == "SCLO" | sub_code == "SFLP" | sub_code == "SSER" | sub_code == "SZIP" | sub_code == "SPAL" | sub_code == "SBRX" | sub_code == "SLUR" | sub_code == "SKRP")|>

group_by(sample_id_month) |>

filter(n() == 1) |>

ungroup() |>

select(sample_id_month)

#Only keep sample_ids that contain measurement of only one antipsychotic

included_mdiff_monotherapy <- included_mdiff |>

filter(sample_id_month %in% monotherapy$sample_id_month)

length(unique(included_mdiff_monotherapy$pat_id))

#Find distribution of dose and concentration in all included TDM samples (for supplement table)

quantile(included_mdiff_monotherapy$mdosis_num, probs = c(0.10, 0.25, 0.50, 0.75, 0.90), na.rm = TRUE)

mean(included_mdiff_monotherapy$mdosis_num, na.rm = TRUE)

sd(included_mdiff_monotherapy$mdosis_num, na.rm = TRUE)

quantile(included_mdiff_monotherapy$conc_num, probs = c(0.10, 0.25, 0.50, 0.75, 0.90))

mean(included_mdiff_monotherapy$conc_num)

sd(included_mdiff_monotherapy$conc_num)

#Remove clozapine samples without corresponding biochemistry

included_mdiff_monotherapy_bc <- included_mdiff_monotherapy_bc |>

filter(!is.na(triglycerides) | !is.na(ldl) | !is.na(hdl) | !is.na(glucose) | !is.na(hba1c))

length(unique(included_mdiff_monotherapy_bc$pat_id))

#Find distibution of dose and concentration in all samples with TDM and MP available (for supplement table)

quantile(included_mdiff_monotherapy_bc$mdosis_num, probs = c(0.10, 0.25, 0.50, 0.75, 0.90), na.rm = TRUE)

mean(included_mdiff_monotherapy_bc$mdosis_num, na.rm = TRUE)

sd(included_mdiff_monotherapy_bc$mdosis_num, na.rm = TRUE)

quantile(included_mdiff_monotherapy_bc$conc_num, probs = c(0.10, 0.25, 0.50, 0.75, 0.90))

mean(included_mdiff_monotherapy_bc$conc_num)

sd(included_mdiff_monotherapy_bc$conc_num)

#Keep only the last sample for each patient

included_mdiff_monotherapy_bc$order_date <- as.Date(included_mdiff_monotherapy_bc$order_date)

complete <- included_mdiff_monotherapy_bc

complete <- complete |>

group_by(pat_id) |>

filter(order_date == max(order_date)) |>

ungroup()

### References in supplements

1.	Wickham, H. (2016) ggplot2: Elegant Graphics for Data Analysis. New York, NY: Springer-Verlag. <https://doi.org/10.1007/978-3-319-24277-4>
